# High-resolution T_2_-weighted cervical cancer imaging: a feasibility study on ultra-high-field 7.0-T MRI with an endorectal monopole antenna

**DOI:** 10.1007/s00330-016-4419-y

**Published:** 2016-05-31

**Authors:** Jacob P. Hoogendam, Irene M. L. Kalleveen, Catalina S. Arteaga de Castro, Alexander J. E. Raaijmakers, René H. M. Verheijen, Maurice A. A. J. van den Bosch, Dennis W. J. Klomp, Ronald P. Zweemer, Wouter B. Veldhuis

**Affiliations:** 1 Department of Gynaecological Oncology, UMC Utrecht Cancer Centre, University Medical Centre Utrecht, PO Box 85500, Heidelberglaan 100, 3584 CX Utrecht, The Netherlands; 2 Department of Radiology, University Medical Centre Utrecht, Heidelberglaan 100, Utrecht, The Netherlands

**Keywords:** Uterine cervical neoplasms, Magnetic resonance imaging, Feasibility studies, Antenna, Neoplasm staging

## Abstract

**Objectives:**

We studied the feasibility of high-resolution T_2_-weighted cervical cancer imaging on an ultra-high-field 7.0-T magnetic resonance imaging (MRI) system using an endorectal antenna of 4.7-mm thickness.

**Methods:**

A feasibility study on 20 stage IB1–IIB cervical cancer patients was conducted. All underwent pre-treatment 1.5-T MRI. At 7.0-T MRI, an external transmit/receive array with seven dipole antennae and a single endorectal monopole receive antenna were used. Discomfort levels were assessed. Following individualised phase-based B_1_^+^ shimming, T_2_-weighted turbo spin echo sequences were completed.

**Results:**

Patients had stage IB1 (*n* = 9), IB2 (*n* = 4), IIA1 (*n* = 1) or IIB (*n* = 6) cervical cancer. Discomfort (ten-point scale) was minimal at placement and removal of the endorectal antenna with a median score of 1 (range, 0–5) and 0 (range, 0–2) respectively. Its use did not result in adverse events or pre-term session discontinuation. To demonstrate feasibility, T_2_-weighted acquisitions from 7.0-T MRI are presented in comparison to 1.5-T MRI. Artefacts on 7.0-T MRI were due to motion, locally destructive B_1_ interference, excessive B_1_ under the external antennae and SENSE reconstruction.

**Conclusions:**

High-resolution T_2_-weighted 7.0-T MRI of stage IB1–IIB cervical cancer is feasible. The addition of an endorectal antenna is well tolerated by patients.

***Key Points*:**

• *High resolution T*_*2*_-*weighted 7.0*-*T MRI of the inner female pelvis is challenging*

• *We demonstrate a feasible approach for T*_*2*_-*weighted 7.0*-*T MRI of cervical cancer*

• *An endorectal monopole receive antenna is well tolerated by participants*

• *The endorectal antenna did not lead to adverse events or session discontinuation*

**Electronic supplementary material:**

The online version of this article (doi:10.1007/s00330-016-4419-y) contains supplementary material, which is available to authorized users.

## Introduction

Accurate staging of cervical cancer is crucial for treatment planning and determines prognosis. Historically, to allow efficient and comparable staging in high incidence underdeveloped areas, the International Federation of Gynaecology and Obstetrics (FIGO) requires clinical (i.e. non-surgical) staging by physical examination [1]. This inherently introduces understaging and overstaging, particularly for intermediate stages wherein estimation of (subtle) parametrial invasion by rectovaginal examination remains difficult, yet determines operability [2]. Studies comparing clinical and post-surgical histological stages in IB1, IB2, IIA1-2 and IIB have reported concordance in 82–85 %, 61–77 %, 35–60 % and 20–59 % of cases, respectively [2–4].

Following the 2009 FIGO update, and supported by (inter)national guidelines, magnetic resonance imaging (MRI) may be added to the work-up to assist clinical staging [5–7]. A meta-analysis (*n* = 3,254, 40 studies) showed a pooled sensitivity of 84 % for detection of parametrial invasion by MRI, substantially superior to the 40 % achieved by clinical examination [8]. This study also identified higher B_0_ field strengths and the use of fast spin echo sequences as statistically significant factors to improve the accuracy in detecting parametrial invasion [8].

Increasing the B_0_ field strength to 7.0 T, increases the signal-to-noise ratio (SNR) and consequently allows for higher spatial or temporal resolution acquisitions [9]. While more expensive, this is potentially advantageous for the assessment of loco-regional invasion which is a predominantly anatomic, spatial resolution-dependent assessment made on T_2_-weighted MR images. Moreover, at 7.0 T, the MRI signals are obtained at much shorter wavelengths than at lower fields, facilitating the use of ultra-thin antennae [10]. While using such an antenna in close proximity to the cervix is more laborious, SNR and thereby resolution is expected to increase even further.

We built an endorectal monopole antenna and aimed to develop dedicated T_2_-weighted TSE sequences for 7.0-T imaging with that antenna combined with an external coil array, to image the (para)cervical anatomy in early stage cervical cancer patients. To date, no published research exists which has attempted this. We assessed patient tolerance of using an endorectal antenna. In addition, we will present the T_2_-weighted images acquired at 7.0 T, and clinical 1.5-T MRI as a visual reference.

## Materials and methods

### Design

We conducted a monocentre, prospective cohort study to develop, optimise and assess the feasibility of high-resolution pelvic T_2_-weighted in vivo imaging on a 7.0-T MRI system using a purpose-designed endorectal antenna. Inclusion criteria were: (1) a histologically proven primary malignancy of the cervix uteri, (2) FIGO stage IB1, IB2, IIA1-2 or IIB disease, and (3) a minimum age of 18 years. Patients were excluded when (1) general contra-indications for MRI existed, (2) radical surgery had already been performed or chemotherapy and/or radiotherapy had been initiated, or (3) uterine prolapse existed (C ≥ −6 cm, POP-Q classification [11]). When eligible, subjects were consecutively counselled between March 2014 and November 2015.

The institutional review board approved this study (clinicaltrials.gov: NCT02083848). Participants provided written informed consent. Data quality, protocol adherence and safety were independently monitored by qualified staff. At our tertiary oncologic referral centre, clinical staging adheres to FIGO and national cervical cancer guidelines [1, 6]. ESM 1 provides details on the clinical 1.5-T MRI and treatment [12].

### 7.0-T MRI

Participants completed a safety checklist and underwent metal detector testing prior to imaging on a whole-body 7.0-T MRI system (Achieva; Philips Medical Systems, Cleveland, USA) equipped with eight-channel multi-transmit functionality. Intravenous contrast agents were not administered, nor was spasmolytic medication. Adverse events were monitored in adherence to the common terminology criteria for adverse events criteria [13].

The shortened B_1_ wavelength at ultra-high-field MRI, which limits signal penetration and increases the risk of destructive interference, challenges cervical cancer imaging given its anatomical position deep in the female inner pelvis. To alleviate these issues, a local transmit/receive array consisting of seven 30-cm fractionated dipole antennae (MR Coils, Drunen, Netherlands) was used. This setup allows for per patient optimisation of the B_1_ field distribution. The technical specifications of this array, including the corresponding specific absorption rate (SAR) implications, were recently published [14].

**Fig. 1:**
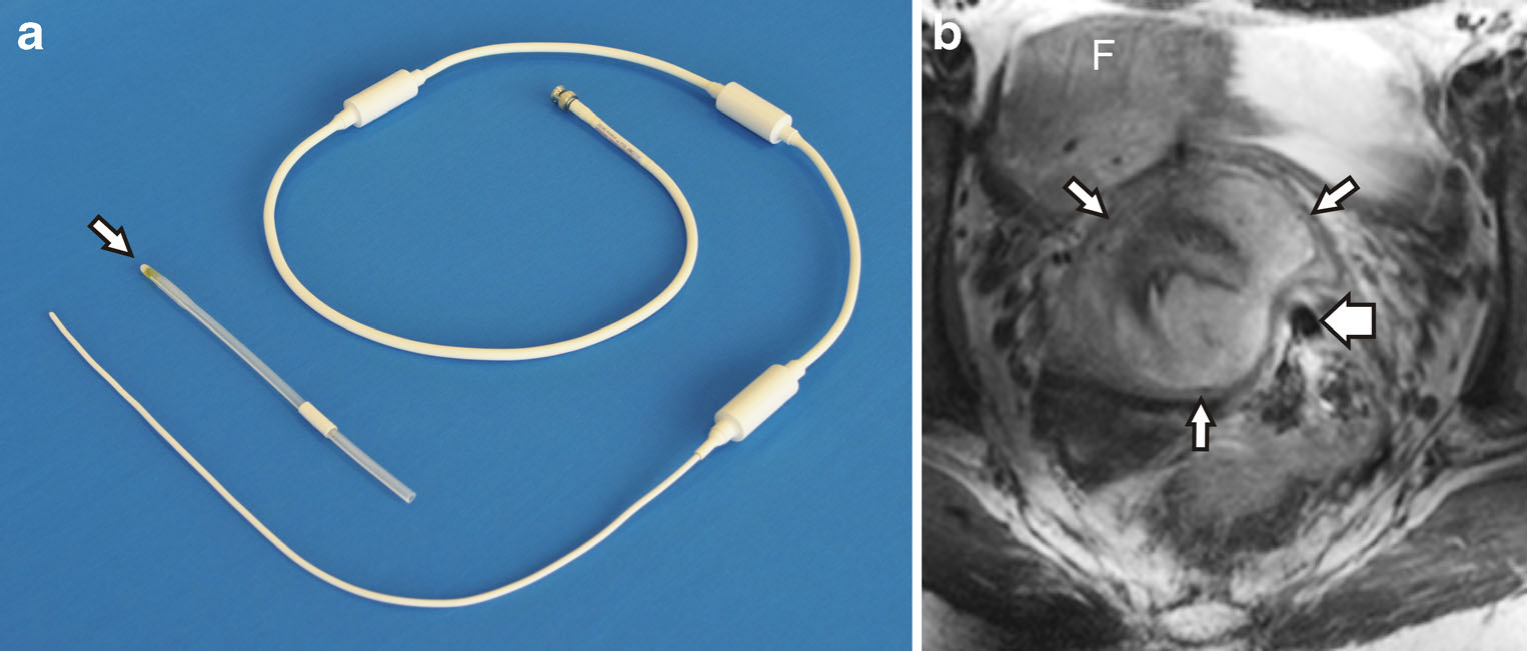
**a** Overview of the monopole antenna shown with the 14-Fr Foley urinary catheter (*arrow*) removed. **b** Transverse T_2_-weighted 7.0-T MRI of the inner female pelvis which demonstrates the close proximity of the endorectal monopole antenna (*broad arrow*) to this stage IB2 poorly differentiated papillary squamotransitional cell carcinoma (*asterisk*) of the cervix. Note the uterine fundus (*F*) and the T_2_ hypointense fibrostromal ring surrounding the tumour (*narrow arrows*) indicative of absent parametrial invasion

The internal monopole B_1_ receive antenna was created in-house and specifically designed for endorectal use in 7.0-T MRI, and subsequently commercialised by Machnet (Maarn, Netherlands). It was positioned in a 14-Fr Foley urinary catheter with a desufflated balloon for an optimal balance between rigidity and flexibility, yielding a 4.7-mm outer diameter (Fig. 1). In addition to its sterilisation in-between sessions, a single-use, sterile cover (Ultracover 200 mm; Microtek Medical, Zutphen, Netherlands) was used. Water-based lubricating gel (K-Y; Johnson & Johnson, Sézanne, France) facilitated easy endorectal positioning. The region with optimal signal strength was located 6–10 cm beyond the anal verge. Patient-reported levels of discomfort related to the antenna—on a Likert scale from 0 (i.e. none whatsoever) to 10 (i.e. worst imaginable)—were assessed directly after introduction and removal.

Sequence parameters were optimised for each patient in the first half of the study. From inclusion 10 and on, a standardised protocol with only minor individual adaptations was used. After a multidirectional survey was obtained for anatomical localisation, phase-based B_1_^+^ shimming was performed per patient to maximise and homogenise the B_1_^+^ on the (para)cervical anatomy [15]. Herein, a single-slice gradient echo sequence was repeated 7 times, each time transmitting with a different transmit antenna, while receiving with all eight antennae. Next, following a shimmed survey, T_2_-weighted TSE sequences in the transverse (repetition time (TR)/echo time (TE) = 7,000/100 ms, radiofrequency (RF) echo train length = 16, flip angle = 90 degrees, matrix = 640 × 640, field of view (FoV) = 250 × 400 × 59 mm, slice thickness/gap = 3/1 mm, duration = 294 s) and sagittal plane (TR/TE = 7,000/100 ms, RF echo train length = 16, flip angle = 90 degrees, matrix = 640 × 640, FoV = 250 × 400 × 73 mm, slice thickness/gap = 3/1 mm, duration = 294 s) were created. Also, a T_2_-weighted TSE axial oblique sequence (TR/TE = 7,000/100 ms, RF echo train length = 16, flip angle = 90 degrees, matrix = 512 × 512, FoV = 350 × 250 × 39 mm, slice thickness/gap = 3/1 mm, duration = 322 s) angled perpendicular to the cervical canal was performed. All T_2_-weighted acquisitions had a voxel size of 0.7 × 0.8 × 3.0 mm and used a SENSE parallel acquisition technique (parallel reduction factor, 3). All sequences remained within the maximum local SAR limit of 10 W/kg [16].

## Results

### Endorectal antenna tolerance

Of the 25 women who waived participation, only one chose not to partake because of objections against the use of the endorectal antenna. In addition to the predetermined sample of 20 patients, three women provided informed consent but could not be imaged due to system unavailability. *See* ESM 2 for the corresponding flowchart. The baseline characteristics of the scanned population are outlined in Table 1.

**Table 1:** Baseline characteristics of the 20 women who underwent 7.0-T MRI

Median age (range)	39.3 (25.3–66.5) years
Median BMI (range)	22.3 (18.4–36.7) kg/m^2^
	*n* (percentage)
Parity	
0	9 (45 %)
1	3 (15 %)
2	8 (40 %)
WHO performance status	
0	17 (85 %)
1	3 (15 %)
ASA classification	
1	13 (65 %)
2	7 (35 %)
Stage	
IB1	9 (45 %)
IB2	4 (20 %)
IIA1	1 (5 %)
IIB	6 (30 %)
Tumour histology	
Squamous cell carcinoma	10 (50 %)
Adenocarcinoma	8 (40 %)
Other	2 (10 %)
Tumour differentiation	
Grade 1	3 (15 %)
Grade 2	8 (40 %)
Grade 3	7 (35 %)
Not applicable	2 (10 %)
LVSI present	5 (25 %)
Lymph node metastases^a^	4 (20 %)
Treatment	
Robot ass. laparoscopic SLN + PLND + RVT or RH	7 (35 %)
Robot ass. laparoscopic SLN + PLND + RH + adjuvant Rth^b^	1 (5 %)
Robot ass. laparoscopic SLN + PLND + chemoradiation^c^	1 (5 %)
PLND + RH via laparotomy^d^	1 (5 %)
Chemoradiation	10 (50 %)

*BMI* body mass index, *WHO* World Health Organisation, *ASA* American Society of Anaesthesiologists, *LVSI* lymphvascular space invasion, *SLN* sentinel lymph node procedure, *PLND* pelvic lymph node dissection, *RVT* radical vaginal trachelectomy, *RH* radical hysterectomy, *Rth* radiotherapy

^a^Determined by a composite of the SLN procedure, PLND or PET-CT as available

^b^Adjuvant radiotherapy was indicated due to a <5-mm resection margin

^c^Chemoradiation substituted radical hysterectomy because of intraoperatively detected tumour-positive sentinel lymph nodes

^d^After diagnosis and staging at our centre, this patient preferred treatment at a different hospital where no laparoscopic radical surgery was performed

Tolerance of the endorectal antenna was excellent, discomfort on the ten-point scale was ‘minimal’ at placement with a median score of 1 (range, 0–5) and reported as ‘none whatsoever’ for removal with a median score of 0 (range, 0–2). The single outlier of 5 at placement occurred in a patient who had undergone ligation of multiple haemorrhoids 1 month earlier. In contrast, a subject with a history of excisional haemorrhoidectomy 4 years earlier had uneventful placement (score, 0) and removal (score, 1). Comparable results were found in cases with irritable bowel syndrome, chronic obstipation and deep infiltrating endometriosis.

None of the participants reported pain or a heating sensation at any time, nor did any subject request preterm termination of the MRI session. The duration in the MRI with the antenna in situ was 48.0 ± 7.3 min. One adverse event—unrelated to the antenna—was reported, namely <30 s of mild vertigo upon entering the 7.0-T MRI bore.

### Cervical cancer imaging

**Fig. 2:**
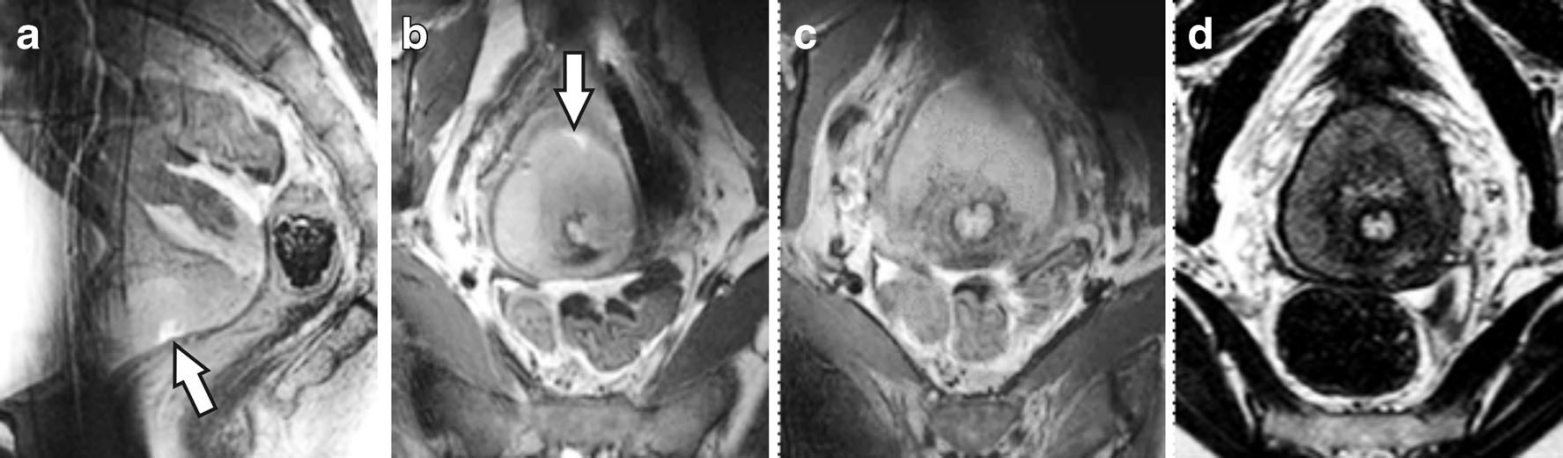
**a** Mid-sagittal and **b** axial oblique (perpendicular to the cervical canal) T_2_-weighted slice at 7.0 T of a 44-year-old patient diagnosed with a 70-mm, stage IB2, poorly differentiated squamous cell carcinoma originating from the ventral part of the cervix. Note the visible biopsy site (*arrow*). **c** Slice from the same sequence, though 12 mm cranially, as **b**, depicting part of the healthy (T_2_ hypointense) cervix invaded by tumour. **d** Axial oblique T_2_-weighted slice from the clinical 1.5-T MRI, created 17 days earlier, matched to **c** for comparison. Note the T_2_ hypointense fibrostromal ring surrounding the tumour

**Fig. 3:**
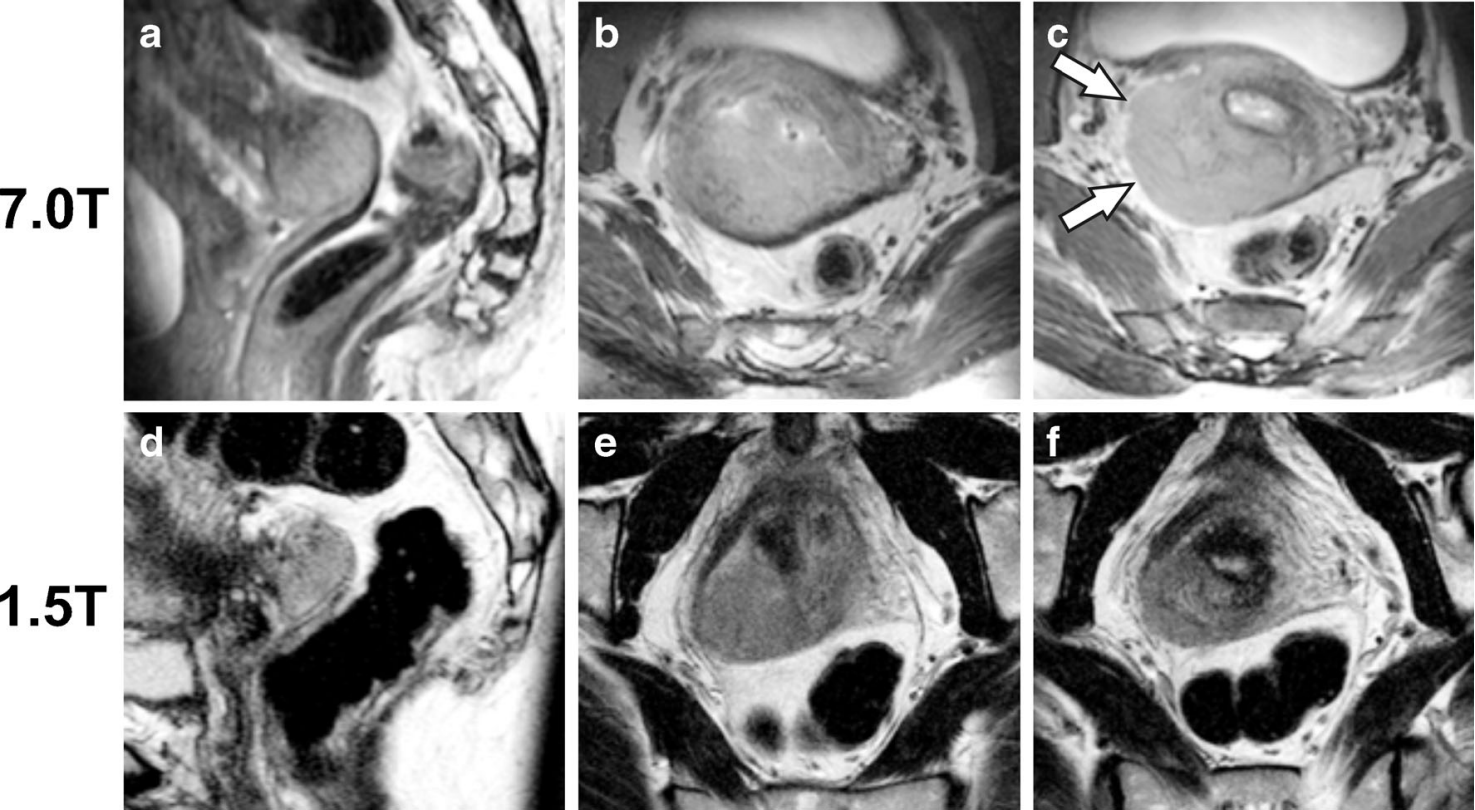
**a** Sagittal and **b** axial oblique T_2_-weighted acquisitions from the 7.0-T MRI of a 48-year-old woman diagnosed with an 80-mm poorly differentiated squamous cell carcinoma of the dorsal cervix. **c**. Slice from the same acquisition as **b**, though positioned 12 mm cranially. Parametrial invasion was judged absent at rectovaginal palpation, leading to a clinical stage IB2. However, the unclear tumour demarcation and absent T_2_ hypointense fibrostromal ring on the right (*arrows*) are suggestive of right-sided parametrial invasion (i.e. stage IIB). **d**-**f** The matched T_2_-weighted axial oblique slices from the clinical 1.5-T MRI, created 24 days earlier, are provided for comparison

**Fig. 4:**
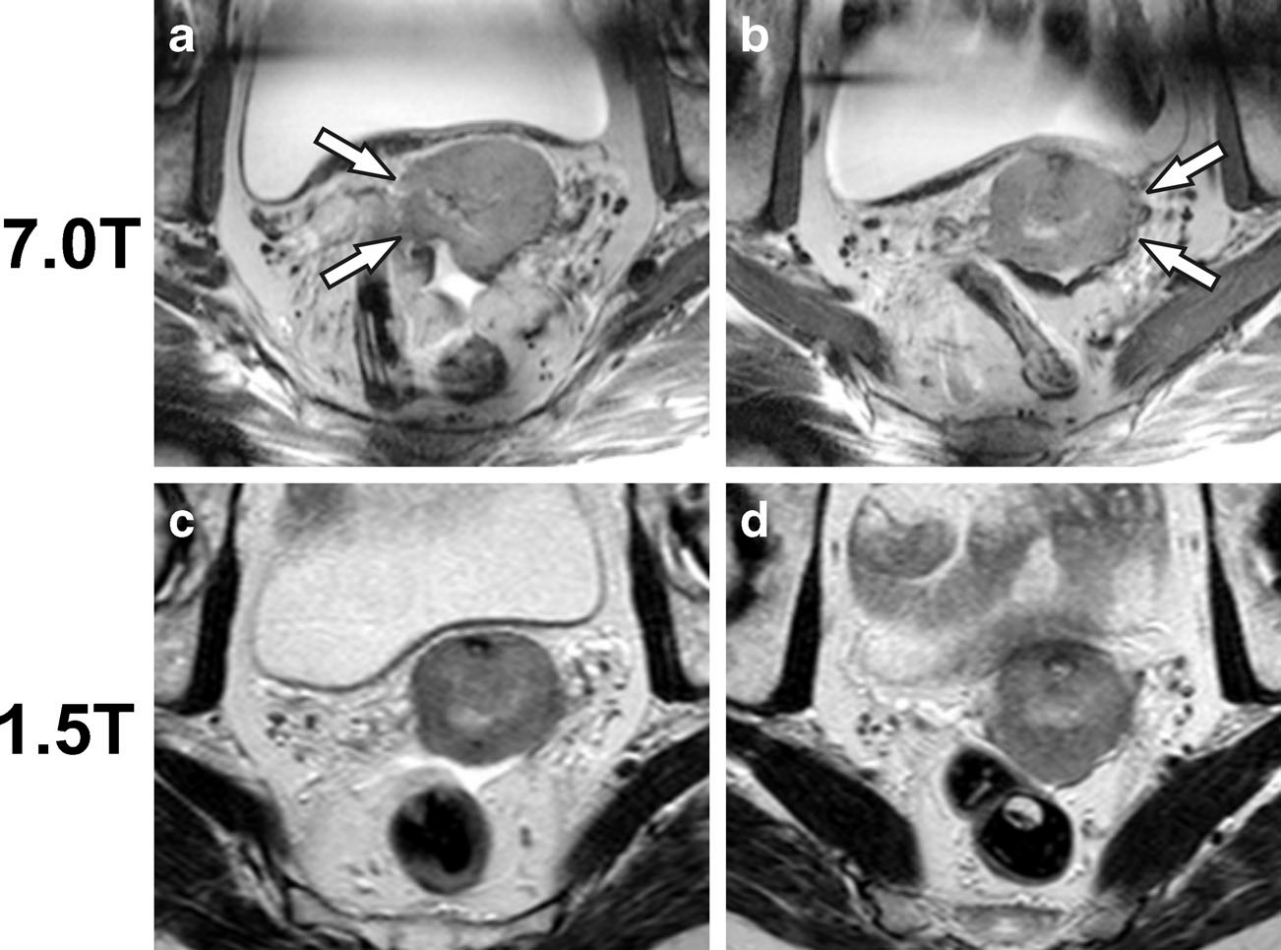
**a** Transverse T_2_-weighted acquisition from the 7.0-T MRI of a 65-year-old woman diagnosed with a 50-mm moderately differentiated squamous cell carcinoma of the cervix. **b** Slice from the same acquisition as **a**, though positioned 8 mm cranially. Only left-sided parametrial invasion was judged present at rectovaginal palpation, leading to a clinical stage IIB. However, the bilaterally unclear tumour demarcation and absent T_2_ hypointense fibrostromal ring are suggestive of bilaterally sided parametrial invasion (*arrows*). **c**, **d** The matched transverse T_2_-weighted slices from the clinical 1.5-T MRI, created 16 days earlier, are provided for comparison. Note the free fluid in the rectouterine pouch (Douglas)

Key to our focus on T_2_-weighted imaging was the visualisation of parametrial invasion, which is particularly challenging when subtle and in large tumours. Here, we present three exemplary cases which represent the range of physical examination and imaging results encountered. First, Fig. 2 presents a woman in whom the physical examination led to a stage IB2, in agreement with 1.5-T and 7.0-T MRI which indicated bilaterally absent parametrial invasion. The second example was clinically staged as IB2, though right-sided parametrial invasion was suspected on both MRIs (Fig. 3). This was motivated by unclear tumour demarcation against the parametrial fat on the right—more distinct on 7.0-T MRI—and a locally interrupted T_2_-hypointense fibrostromal ring. The third example was a bulky IIB based on left sided parametrial invasion at rectovaginal examination. However, the 7.0-T MRI was considered suggestive of bilateral parametrial invasion (Fig. 4). All three cases received chemoradiation, hence no definitive histological proof of invasion was provided. The mean interval between the clinical 1.5-T and experimental 7.0-T MRI was 13.7 ± 11.8 days. None of the nine included women with a clinical stage IB1 tumour had an unexpected histological finding of parametrial invasion following their radical surgery.

A prior loop excision, sharp conisation or both were performed in three, one and two women, respectively. The interval of this surgery to the clinical 1.5-T and 7.0-T MRI was a median 42 days (range, 32–44 days) and 47 days (range, 41–57 days) respectively. After radical surgery, final histology did not show residual invasive tumour in any of these cases.

### Artefacts

**Fig. 5:**
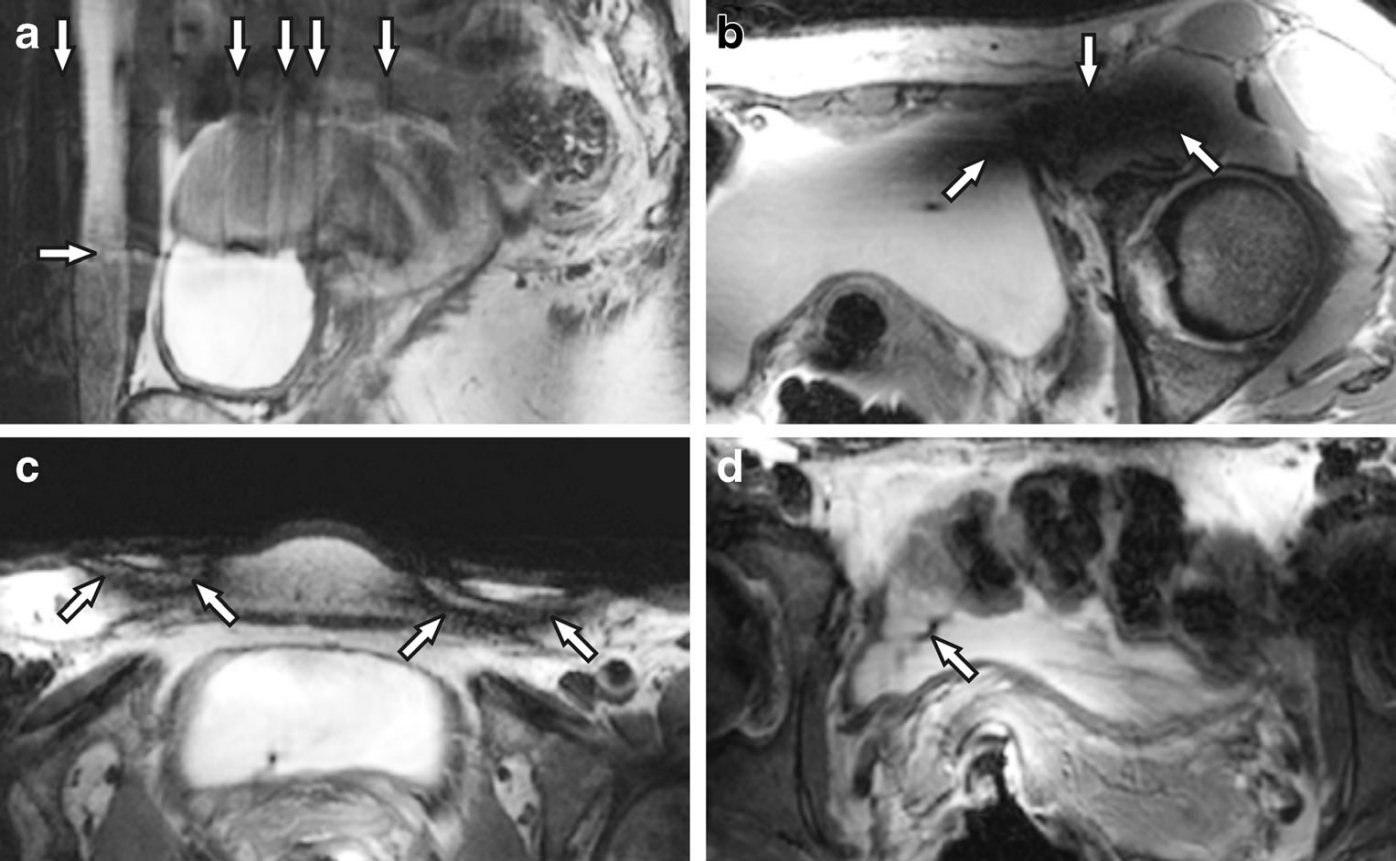
Image artefacts that were encountered on 7.0-T MRI were **a** motion artefacts, **b** locally destructive B_1_ interference, **c** inversion bands due to too much B_1_ under the external transmit/receive antennae and **d** SENSE reconstruction artefacts. Note the unrelated vaginal tampon (*asterisk*) in **c**

On sagittal acquisitions, motion artefacts in the phase encoding direction, caused by breathing, occurred relatively frequently (Fig. 5a). Secondly, non-essential anatomical regions were variably obscured by signal voids caused by destructive interference of B_1_—due to the short RF wavelength at 7.0 T—from the multiple independent external transmit antennae (Fig. 5b). Thirdly, superficial black semicircular inversion bands were present due to the inherently much higher B_1_ levels directly under the elements of the external transmit/receive antenna array (Fig. 5c). While encountered in all participants, it posed no clinical problem as only the subcutaneous fat was obscured. Fourthly, small SENSE reconstruction artefacts were incidentally seen, and are likely caused by destructive interference in the receive signals of the SENSE reference scan (Fig. 5d).

## Discussion

This feasibility study showed that T_2_-weighted cervical cancer imaging at 7.0 T is achievable and that the incorporation of an endorectal antenna is well tolerated by patients. We have presented the acquired images, referenced against 1.5-T MRI, relevant for local tumour assessment. To our knowledge, no literature currently exists on 7.0-T MRI in cervical cancer, which in the past has been termed ‘a considerable challenge’ [17]. The presented study demonstrates a feasible approach to body imaging for pathology in the female pelvis.

Earlier research on 7.0-T MRI in the female pelvis was obtained with an external coil array only, limited to healthy volunteers and reported moderate image quality of T_2_-weighted sequences [18]. Our approach incorporated an endorectal monopole antenna for optimal signal capture, improving the SNR, deep in the inner pelvis [19]. Its use was not judged as uncomfortable, nor did it prohibit study accrual. Furthermore, in our small sample, no adverse events related to the antenna were encountered.

The research group led by Nandita deSouza has published extensively on their in-house built 37-mm ring-shaped solenoid receive coil, placed endovaginally around the cervix, for 0.5- to 3.0-T MRI in stage IA, IB1 and IIA cervical cancer [20, 21]. Its application appears limited to relatively small lesions, though accurate in tumour detection and volume calculation [22–24]. Unfortunately, for parametrial invasion detection on T_2_-weighted imaging no conclusions have thus far been reached on the added value of this solenoid receive coil [25]. In a recent study on radical surgery (*n* = 25), only one patient had unexpected parametrial extension which was missed on MRI with the solenoid receive coil [25].

In line with the above, a limitation of our study is that none of the women clinically suspected of parametrial invasion had histological confirmation. The risk of partial verification bias is inherent to current practice guidelines, which preclude radical surgery for women with tumour extension outside the cervix [6, 7, 26]. While definitive proof would have strengthened our case presentation, this was prohibited by the inherent design of our study which was not aimed at diagnostic accuracy.

Several technical challenges in our study on pelvic imaging at ultra-high field strength merit further explanation. The SNR advantage of the endorectal antenna is local, which limits the high-resolution field of view in the feet-head direction and does not—for example—permit enhanced visualisation of lymph nodes at the common iliac arteries [19]. While relevant for a clinical MRI protocol, this was not an objective of the current study, which focused on the feasibility of primary tumour imaging. Secondly, at ultra-high field strengths the tissue RF power deposition is substantial and, in RF pulse intensive sequences like TSE used for T_2_-weighted imaging, leads to SAR constraints. As a consequence, the repetition time has to be increased, which lengthens the scan protocol. Internal antennae may, however, alleviate this by taking advantage of its highly non-uniform spatial field distribution that can be used for zoomed imaging or high imaging accelerations [14]. In addition, the short B_1_ wavelength at ultra-high field strengths causes B_1_ inhomogeneity and destructive interference, yielding artefacts which may obscure relevant parts of the inner pelvic anatomy. Using multi-dimensional RF pulses, these artefacts may be removed [27]. Our individualised B_1_ shimming approach, made possible by using an external body array coil with multiple elements in parallel transmission, ensured that key anatomical regions of interest (i.e. the cervix) remained visible. Finally, the SENSE reconstruction algorithm that was implemented by the manufacturer, uses at the time of the study a reference scan with a constant amplitude and phase weighting during reception. This can cause destructive interferences during reception, causing artefacts (Fig. 5d). These artefacts can be mitigated using interferometry techniques [28].

Future studies should focus on whether our experimental imaging technique improves clinical decision making. This includes quantifying both the diagnostic test accuracy and observer variability (i.e. reproducibility). Furthermore, we focused on T_2_-weighted imaging as it is relevant for local tumour assessment, though for clinical implementation additional sequences such as T_1_-weighted MRI are desired [29]. The addition of functional imaging such as ^1^H or ^31^P MR spectroscopy—current experience in cervical cancer is limited to 1.5- to 3.0-T MRI—may benefit from the increased spectral and spatial resolution at ultra-high B_0_ field strengths [30, 31].

In conclusion, the use of an endorectal monopole antenna to improve the SNR at the level of the cervix was well tolerated by participants and not associated with any real discomfort, nor did it lead to adverse events or hinder study accrual. We established the feasibility of T_2_-weighted cervical cancer imaging with 7.0-T MRI. While further research is needed to reduce artefacts and substantiate its clinical impact, we demonstrated that high-resolution T_2_-weighted acquisitions deep in the female pelvis can be achieved with ultra-high-field MRI. This combination of ultra-high-field MRI and an internal antenna is promising and merits further research, including pelvic imaging for indications beyond cervical cancer.

## Electronic supplementary material

Below is the link to the electronic supplementary material.

ESM 1 Standard clinical care, including 1.5-T MRI sequence protocol (DOCX 13 kb)

ESM 2 Flowchart of patient accrual into the study (DOCX 25 kb)
