# Supplementary material for: High-resolution T2-weighted cervical cancer imaging: a feasibility study on ultra-high-field 7.0-T MRI with an endorectal monopole antenna
Source: Eur Radiol. 2016 May 31;27(3):938–45. doi: 10.1007/s00330-016-4419-y (PMC5306309; doi:10.1007/s00330-016-4419-y)
Supplement: Supplementary file 1 — Standard clinical care, including 1.5-T MRI sequence protocol (DOCX 13 kb) [file 330_2016_4419_MOESM1_ESM.docx]

**Supplemental file 1: Standard clinical care**

At our tertiary referral centre, clinical staging adheres to FIGO and national cervical cancer guidelines. This includes a re-examination under anaesthesia when the outpatient exam is deemed or unreliable. All patients routinely undergo pelvic MRI on a 1.5T system (Achieva/Intera, Philips Medical systems, Best, the Netherlands) with an external sensitivity encoding (SENSE) torso coil only. The protocol consists of a transversal *T*_2_-weighted TSE sequence (repetition time (TR) / echo time (TE)=6667/100ms, flip angle=90 degrees, matrix=400x280, Field of View (FoV)=320x320mm, slice thickness/gap=4/0mm), a sagittal *T*_2_-weighted TSE sequence (TR/TE=2800/100ms, flip angle=90 degrees, matrix=424x280, FoV=300x240mm, slice thickness/gap=4/0mm) and a double-oblique *T*_2_-weighted TSE sequence angled perpendicular to the cervical canal (TR/TE=3030/100ms, flip angle=90 degrees, matrix=400x280, FoV=320x320mm, slice thickness/gap=4/0mm). In addition, a transversal diffusion-weighted sequence (*b*-values: 0 and 800 s/mm^2^), a transversal fat-saturated *T*_1_-weighted sequence before and after intravenous gadolinium, and a transversal proton-density-weighted sequence of the entire abdomen were performed. In cases where a diathermic loop excision or a cold knife cone had been performed the clinical MRI was scheduled at least 30 days after such procedure to minimize tissue reaction, which may obscure residual tumour.

Stage IB1 and IIA1 patients were scheduled for a sentinel lymph node procedure with frozen section, pelvic lymph node dissection and radical hysterectomy or – in eligible patients desiring fertility preservation – a radical vaginal trachelectomy. In patients with tumour-positive (sentinel) lymph nodes, chemoradiation substituted radical uterine surgery. Stage IB2, IIA2 and IIB patients were treated primarily with chemoradiation. By design, treatment and stage decisions were uninfluenced by 7.0T MRI findings, which were not reported to clinicians.
