# Supplementary material for: High-resolution T2-weighted cervical cancer imaging: a feasibility study on ultra-high-field 7.0-T MRI with an endorectal monopole antenna
Source: Eur Radiol. 2016 May 31;27(3):938–45. doi: 10.1007/s00330-016-4419-y (PMC5306309; doi:10.1007/s00330-016-4419-y)
Supplement: Supplementary file 2 — Flowchart of patient accrual into the study (DOCX 25 kb) [file 330_2016_4419_MOESM2_ESM.docx]

**Supplemental file 2:** Flowchart of patient accrual into the study.

48 women were

eligible and counselled

23 women provided

informed consent

20 women completed

7.0T MRI with antenna

25 women waived participation:

- Too overwhelmed by cancer diagnosis, n= 13

- Claustrophobia / finds MRI unpleasant, n= 8

- No reason specified, n=2

- Objection to use of endorectal antenna, n= 1

- Influenza on day of MRI, n= 1

3 women dropped out of the study:

- MRI unavailable (unrelated to study), n= 3

11 women treated with chemoradiation

9 women treated with radical surgery
